# Romosozumab significantly improves vertebral cortical bone mass and structure compared with teriparatide, whereas both treatments increase vertebral trabecular bone mass similarly: high-resolution quantitative computed tomography analyses of randomized controlled trial results in postmenopausal women with low bone mineral density

**DOI:** 10.1093/jbmrpl/ziaf119

**Published:** 2025-07-15

**Authors:** Timo Damm, Cesar Libanati, Jaime Peña, David A Hanley, Michael A Bolognese, Christopher Recknor, Claus-Christian Glüer

**Affiliations:** Department of Radiology and Neuroradiology, Section Biomedical Imaging, Christian-Albrechts-Universität zu Kiel, Kiel, 24118, Germany; UCB, Brussels, 1070, Belgium; Department of Radiology and Neuroradiology, Section Biomedical Imaging, Christian-Albrechts-Universität zu Kiel, Kiel, 24118, Germany; Department of Medicine, University of Calgary, Calgary, AB T2N 4N1, Canada; Bethesda Health Research Center, Bethesda, MD 20814, United States; United Osteoporosis Centers, Gainesville, GA 30501, United States; Department of Radiology and Neuroradiology, Section Biomedical Imaging, Christian-Albrechts-Universität zu Kiel, Kiel, 24118, Germany

**Keywords:** romosozumab, HR-QCT, osteoporosis, clinical trials, BMD

## Abstract

Romosozumab, a sclerostin inhibitor, exerts a dual effect of increasing bone formation while decreasing bone resorption to rapidly increase bone mineral density (BMD) and reduce fracture risk in postmenopausal women. Iterative Convolution OptimizatioN (ICON) allows accurate calculation of deconvolved cortical thickness from high-resolution quantitative computed tomography (HR-QCT) scanning. This study employed HR-QCT to evaluate compartmental (including endosteal and periosteal) changes in the vertebral cortical shell in postmenopausal women who received romosozumab, teriparatide, or placebo. In a subset of a phase 2, randomized study (NCT00896532), women (55-85 yr) with low BMD (T-score ≤ −2.0, but not <−3.5, at the lumbar spine, total hip, or femoral neck) treated with subcutaneous (SC) romosozumab monthly (210 mg; *n* = 11), SC teriparatide daily (20 μg; *n* = 12), or SC placebo (*n* = 8) had spine HR-QCT scans at baseline and Month 12 to assess treatment effects on cortical and cancellous compartments of the T12 vertebra. HR-QCT was obtained at 120 kVp and 360 mAs. Cortical changes were evaluated using ICON software. At Month 12, compared to teriparatide and placebo, romosozumab treatment was associated with greater gains in all cortical parameters. Changes in cancellous bone parameters were similar with romosozumab and teriparatide. Romosozumab significantly increased cortical thickness (mean ± SD; 53 ± 18%) and the magnitude of this change was greater than that of teriparatide (20 ± 13%) and placebo (3 ± 6%); all *p* < .001. With romosozumab, cortical BMC and apparent cortical BMD were also significantly increased from baseline, and compared to teriparatide and placebo (all *p* < .001). These changes occurred through endosteal and periosteal bone matrix apposition, with greatest changes seen at the endosteal surface. The location and magnitude of these changes likely form the basis of the rapid improvement in bone mass, structure, and strength that contribute to romosozumab’s rapid vertebral fracture risk reduction efficacy; however, conclusions are limited by the small sample size.

## Introduction

Osteoporosis is characterized by low bone mass and defects in microarchitecture, which culminate in compromised bone strength and an increased risk of fractures.^1^ The majority of patients suffering from osteoporosis are treated with antiresorptive agents, which may increase volumetric bone mineral density (BMD) and curtail structural damage, but do not stimulate bone formation. Treatment with antiresorptive agents do not lead to optimal outcomes for some patients, particularly those at very high fracture risk.^2^^,^^3^

Sclerostin inhibition increases bone formation and also reduces bone resorption.^4^ In a 1-yr phase 2 clinical trial (NCT00896532), the sclerostin inhibitor romosozumab increased BMD in postmenopausal women with low areal BMD (aBMD) and was associated with increased bone formation and decreased bone resorption.^5^ The antifracture efficacy of romosozumab, mediated by rapid bone mass accrual and structural improvements, has been demonstrated in large phase 3 clinical trials.^6–9^

Vertebral strength is determined by vertebral BMD and volume, as well as by microarchitectural characteristics, such as trabecular structure and cortical thickness.^10–12^ Cortical thickness at the vertebral bodies typically ranges from ~200 to 600 μm, with mean values between ~200 and 400 μm, similar to the thickness of an eggshell, but can be markedly reduced in patients with osteoporosis.^13–16^ Cortical bone has an outer periosteal surface and an inner endosteal surface. However, the contribution of endosteal vs periosteal changes, as well as cortical porosity in the vertebral cortical shell, and the effects of osteoanabolic therapies on endosteal and periosteal apposition in patients with osteoporosis, has not been studied in patients undergoing treatment.

High-resolution quantitative computed tomography (HR-QCT) enables non-invasive measurement of volumetric BMD and assessment of the cortical and trabecular bone separately.^17–19^ Direct measurement of cortical thickness via typical CT scanning can result in substantial overestimation.^20^ Numerous methods have been developed to refine estimations of cortical and trabecular parameters, such as threshold-based approaches and the full-width half-maximum method.^21^^,^^22^ Advances in image processing methods, such as the deconvolution-based method of Treece et al., which applies assumptions about the shape of the cortex, are increasingly able to mitigate such limitations.^23^ The use of HR-QCT scanning further offers more accurate resolution of bone changes compared to the standard QCT method employed by Poole et al., due to its higher spatial resolution and smaller field of view. In turn, this allows for a more detailed visualization of bone microarchitecture, which is important for assessing bone health and fracture risk.

By applying the Iterative Convolution OptimizatioN (ICON) method, a measure of deconvolved cortical thickness (whereby the convolution effects during data acquisition, reconstruction, and processing are estimated and corrected for), adjusted for partial volume effects, can be derived from both standard QCT and HR-QCT scanning protocols using clinical whole-body CT scanners; it is also possible to derive a measure of cortical BMD.^20^ While cortical bone mapping generates 3D maps of thickness by averaging over “local neighborhoods” to generate the mapping voxels, the ICON method can achieve stable estimates of cortical apposition at very thin cortices and detect thickness changes far below the dataset’s voxel size by carrying out a model fit procedure on all data collected around the vertical cortex at once.^24^ In doing so, the ICON method reduces overestimation bias and produces more accurate estimates of cortical thickness that correlate with gold-standard high resolution peripheral QCT (HR-pQCT) measurements.

Reporting on the effects of 12 mo of romosozumab or teriparatide treatment on the first lumbar vertebra (L1) of postmenopausal women with low bone mass in a phase 2, placebo-controlled trial, Poole et al. demonstrated substantial improvements in standard QCT-measured cortical and cancellous bone parameters, which were significantly greater with romosozumab than teriparatide.^5^^,^^25^ However, the method of Poole et al. was not sufficiently sensitive to determine the presence or absence of periosteal apposition in response to treatments. In the present study, using cortical shell segmentation-based layering of HR-QCT scans of thoracic vertebral bodies (T12), we evaluated compartment-specific changes in BMD in response to romosozumab, teriparatide, and placebo administration in the same phase 2 study. By applying a deconvolution-motivated fitting procedure, we assessed changes in BMD and bone mineral content (BMC) in vertebral bone compartments, as well as in microstructural parameters of the vertebral cortical shell.

The purpose of this study was to use HR-QCT to expand upon the work of Poole et al. and evaluate compartmental changes in bone parameters, including changes at the subcortical endosteum, the central cortex, and the periosteum within the vertebral cortical shell in a subset of postmenopausal women with low BMD who received romosozumab, teriparatide, or placebo in a phase 2 randomized trial.^5^^,^^25^

## Materials and methods

### Study design

This analysis utilized data from a completed phase 2, multicenter, international, randomized, placebo-controlled study (NCT00896532) of postmenopausal women aged 55-85 yr with low BMD (a T-score of −2.0 or less at the lumbar spine, total hip, or femoral neck, but not lower than −3.5 at any of the 3 sites). The full study design and main results have been previously reported.^5^ The study protocol was approved by an independent ethics committee or institutional review board at each study site before commencement, and was in accordance with the ethical standards of the Helsinki Declaration. All participants provided written, informed consent prior to participation.

Participants were randomly assigned to receive subcutaneous (SC) romosozumab monthly (QM; 210 mg, *N* = 50), SC teriparatide daily (QD; 20 μg, *N* = 49), or SC placebo (*N* = 50; randomly assigned to QM or Q3M). All participants were required to take at least 1000 mg of elemental calcium and 800 international units (IU) of vitamin D, daily.

aBMD was assessed using Dual X-ray absorptiometry (DXA). A subset of study participants received HR-QCT scans of T12 (*n* = 11 romosozumab, *n* = 12 teriparatide, *n* = 8 placebo [QM: *n* = 2; Q3M: *n* = 6]) at baseline and Month 12. The T12 vertebra was chosen because the long axis of the vertebra is typically well-aligned with the long axis of the scanner, thus reducing the distortions of cruder resolution in the z axis.

Target scans (0.18-0.23 mm in-plane and 0.30 mm out-of-plane pixel size; 120 × 120 × 86 mm^3^ field of view [FOV]) were calibrated using a large FOV scan (0.70 mm in-plane and 0.62 mm out-of-plane pixel size; 360 × 360 × 86 mm^3^ FOV) including a Mindways calibration phantom. Scans were performed using a tube voltage of 120 kVp and an exposure of 360 mAs; however, slight variations in scan parameters were introduced due to the use of scanners from different manufacturers and thus different kernels. Data were collected on Siemens Somatom AS (slice thickness: 1.00 mm; *n* = 4; kernel B70s), Philips Brilliance 64 (slice thickness: 1.00 mm; *n* = 7; kernel D) and GE Medical Systems Lightspeed 16 (slice thickness: 1.25 mm; *n* = 20; kernel BONE) CT scanners.

### HR-QCT scan analysis

Standard cancellous bone compartment variables, previously described,^19^ were evaluated using HR-QCT. Standard volumes of interest (VOIs) for analysis of the densitometric and structural parameters of the vertebrae were generated after carrying out a template-driven, semi-automatic, active shape segmentation approach (a well-established method in CT imaging to delineate structures through iterative adjustment based on learned statistical constraints)^26–28^ with the in-house software Structural*Insight*.^29^ After automatically fitting a mesh onto the thin vertebral cortex, a user-interactive correction was carried out. Afterwards, global and local threshold techniques were used to expand the segmentation outwards to the periosteal, and inwards to the endosteal, cortical borders. Moreover, the cortical segmentation, as well as the enclosed inner trabecular region, were subdivided into several VOIs using a region template.

Cancellous and cortical compartment variables (previously described)^19^^,^^20^^,^^30^ were evaluated within these VOIs, including total trabecular volume, ellipse, vertical cortex, upper end plate, lower end plate, foramen, and cut pedicles.

The mean treatment effect of a bone-forming agent was expected to result in additional matrix deposited on the endosteal and/or periosteal surface of the cortical shell. These structures appear blurred due to limited spatial resolution of the imaging CT system and by subsequent data processing steps as part of the reconstruction kernel. This complete imaging process can be well approximated by assuming a Gaussian point spread function (PSF) as a convolution kernel.^22^ An outline of the mathematical modeling employed in the analyses reported in this manuscript is provided in Supplementary Material S1**.**

Depending on changes in the bone resorption rate, some increases in porosity or reductions in tissue mineral density (TMD) may be induced in the cortex present at baseline; the impact of which will affect (i.e. reduce) measured changes in the endosteal or periosteal zones due to the limited spatial resolution.

For cortical HR-QCT analysis, nested 200 μm thin 3D layers adjacent to an initial cortical shell segmentation, each with a particular constant distance range to it, were evaluated to compute radial density profiles. Hence, changes in BMD and BMC from the outer soft tissue bordering the vertebrae over the cortex itself into the medullary spongiosa were determined. For improved accuracy, spongiosa and cortical regions were decomposed in a mathematical model. Briefly, a spongiosa plateau of variable density, represented mathematically by an edge spread function (ESF), was modeled adjacent to a cortical model, represented by a line spread function (LSF), as described in Supplementary Material S1. Using this model setup for the fit procedure of the complete radial BMD profile, subtraction of the spongiosa plateau’s signal from the cortex becomes feasible, enabling improved detection of cortical apposition.

**Figure 1:**
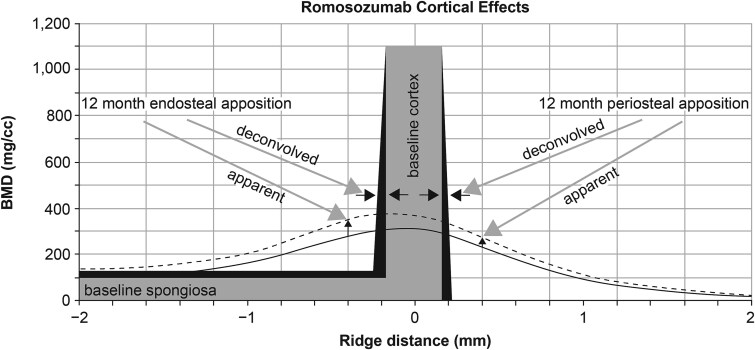
Schematic of bone apposition. An analytically derived, deconvolution-motivated model^22^^,^^31^ (light gray) was used for baseline analysis. For assessing apposition, the model was refined by a specific apposition scheme (black). As a representative example, the averaged radial profiles of the romosozumab-treated group (*n* = 11) are shown at baseline (solid line) and at Month 12 (dashed line).

Active shape segmentation was used for initial periosteal and endosteal segmentation (structure delineation) of vertebral bodies. Cortical thickness (Ct.Th) and density-weighted cortical thickness (wCt.Th) were then calculated based upon these segmentation borders. Moreover, we determined deconvolved cortical thickness (dcCt.Th) using the ICON technique, which has been validated in embedded vertebral phantoms,^20^ and is schematically shown in Figure 1.

Ct.Th was calculated for the vertical cortex only by applying the standard Maximum Sphere algorithm.^32^ Briefly, the cortical segmentation was filled with simulated spheres that touched both boundaries of the periosteum and endosteum, respectively. All voxels (volume elements that define a point in 3D space) included in the structure were labeled with the maximum diameter of a sphere centered at the voxel and contained within the given structure.^20^ In addition to the simple mean value of these “diameter” labels, 2 further measures of cortical thickness were derived (wCt.Th and dcCt.Th).

wCt.Th was determined by multiplying the “diameter” labels forming standard Ct.Th by the factor BMD/1100 mg per cubic centimeter of calcium hydroxyapatite (mgCaHA)/cc. Hence, this corrected the partial volume effect when assuming a fully mineralized cortex as the dominant structure depicted within the cortical segmentation.

dcCt.Th reflects a further refined thickness measure using the ICON method, where density profiles perpendicular to the cortex were collected around the vertical cortex and analyzed in a forward-modeling approach incorporating a combined trabecular-cortical model.

At baseline, a model of cortical compact mineral bone, with an initial width calculated from wCt.Th and an assumed fixed mineralization of 1100 mgCaHA/cc; (in agreement with the physiological range of TMD in fully mineralized human bone),^33^ was fitted via scanner-specific PSF to the baseline density profile in a nonlinear forward-modeling process. This process simultaneously incorporated the fit of the spongiosa plateau.

Changes in cortical thickness from this baseline model were then modeled assuming an average of 50% mineralization, to compensate for newly added matrix still to be mineralized in the same way. In contrast to cortical thickness maps,^23^^,^^34^ BMD, BMC, and Ct.Th were assessed on density profiles, collecting data layer by layer, to minimize the influence of noise, which results in high signal stability in exchange for topology information. Due to the resolution in imaging, both BMD and BMC measurements inevitably underestimate the true values. Therefore, “apparent” cortical BMC (the sum of all voxel volumes multiplied by their respective BMD within the cortical segmentation) and apparent BMD are reported.

Cancellous bone parameters were also evaluated using Structural*Insight*. The threshold-based parameters total bone volume relative to total tissue volume (BV/TV) and TMD, were measured after first applying a threshold of 250 mg/cc to the data. TMD was determined by quantifying the mean density of all voxels above the threshold within the trabecular segmentation. BV/TV was calculated as the fraction of those voxels above the threshold divided by all voxels within the trabecular segmentation.

All image processing and analysis was performed blinded to treatment.

### Statistical analysis

All statistical analyses were performed with JMP Pro 16.0.0 (SAS Institute Inc., Cary, NC, USA). Following one-way analysis of variance (ANOVA), significant differences between treatment arms, and significant changes from baseline to Month 12 within treatment arms, were detected using student’s *t*-tests (using a threshold of *p* < .05) for all measured parameters.

## Results

**Figure 2:**
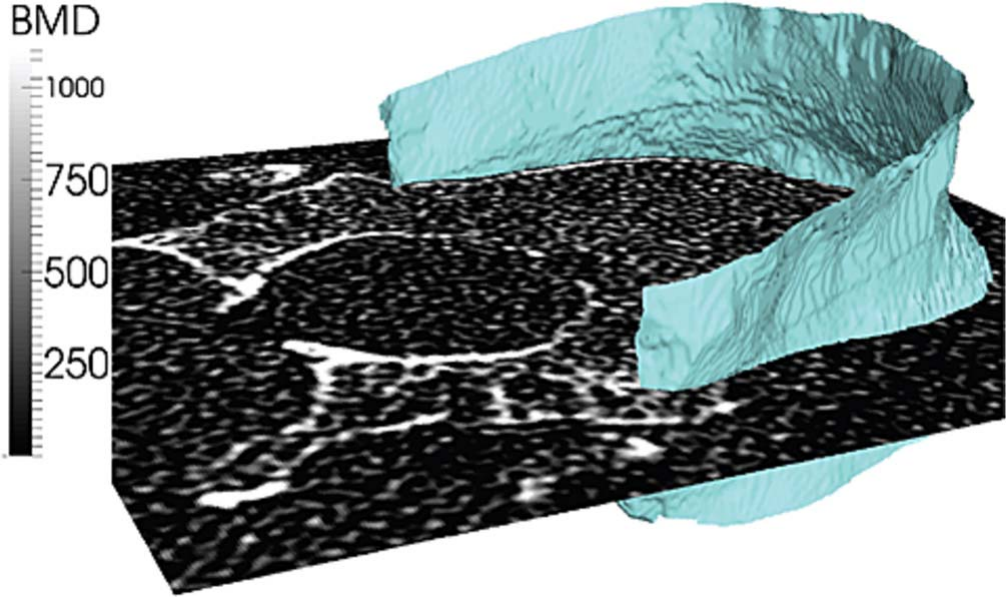
Representative vertebral cortex demonstrating the narrow cortical thickness as calculated for the parameter cortical thickness (Ct.Th; clipped for visualization). Abbreviations: BMD, bone mineral density; Ct.Th, segmentation-based cortical thickness.

A representative example of the thin structure of the cortex, after applying the deconvolution-motivated correction ICON procedure, positioned on an axial slide showing BMD in gray levels is illustrated in Figure 2. At baseline, mean ± SD apparent cortical thickness of 1280 ± 104 μm was corrected to a cortical thickness of 359 ± 52 μm with scanner/reconstruction-kernel-specific PSF (Gaussian parameters [Siemens/B70s: 0.39; Philips/D: 0.61; GE Medical Systems/BONE: 0.55] were estimated from the data to compensate for scanner-/reconstruction-kernel-specific variations).

**Figure 3:**
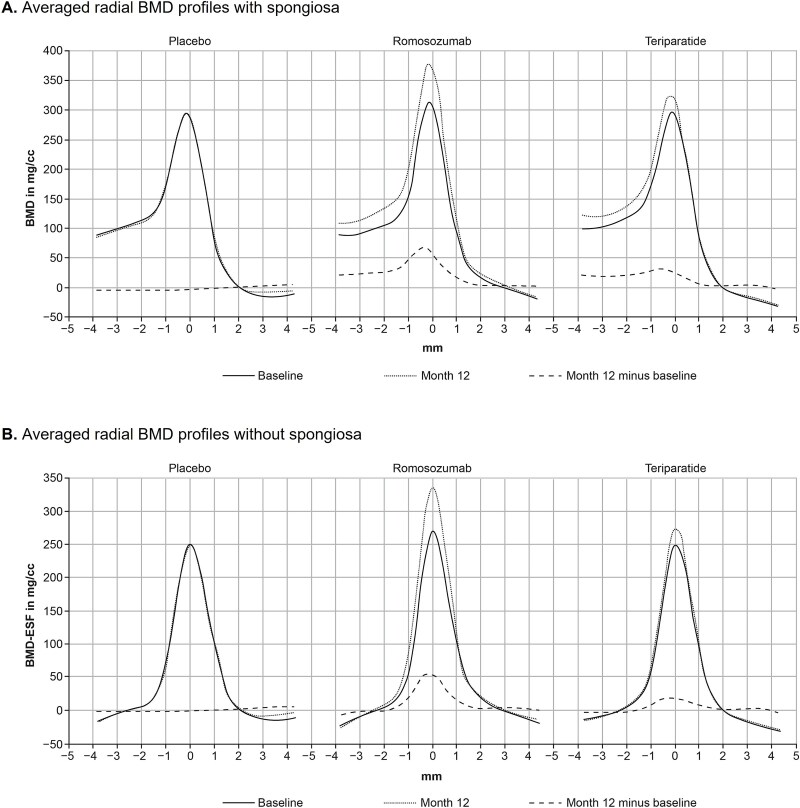
Averaged vertebral cortical BMD (mg/cc) over radial distance (mm) profiles grouped by treatment before (A) and after (B) subtraction of the contribution of spongiosa. Undisturbed spongiosa BMD level (spongiosa plateau) was estimated at some distance to the cortex. Using the obtained plateau height, an ESF was subtracted from the data using σ, as estimated during the fit procedure for the complete radial BMD profile. Abbreviations: BL, baseline; BMD, bone mineral density; ESF, edge-spread function.

The radial BMD profile, averaged layer by layer and across all patients per group, is plotted before (solid line) and after (dotted line) treatment, with subtracted residual signal (dashed line), demonstrating the density changes over 12 mo (Figure 3). Averaged radial BMD profiles are presented both before (Figure 3A) and after (Figure 3B) the contribution of spongiosa (plateau) was subtracted to collect cortical information unbiased by differences in spongiosa changes.

**Figure 4:**
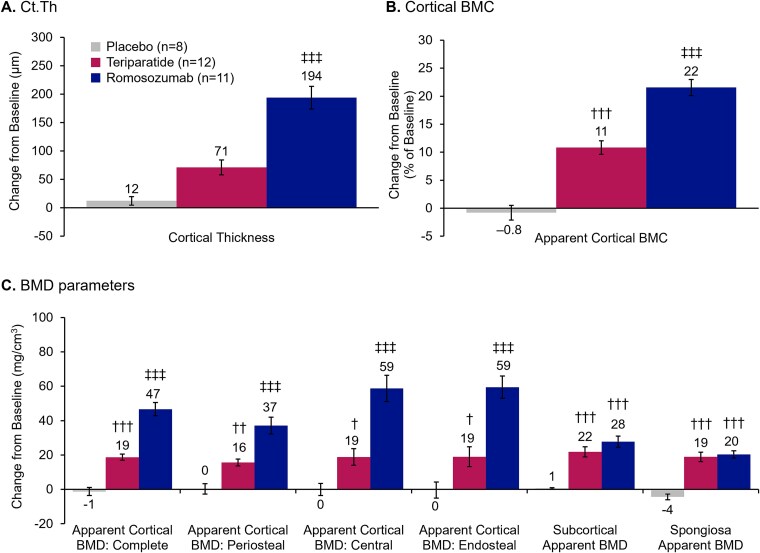
Changes from baseline in vertebral Ct.Th, cortical BMC, and apparent BMD at Month 12. Values are mean ± SEM. Significantly different from placebo: ^†^*p* < .05; ^††^*p* < .01; ^†††^*p* < .001; significantly different from both placebo and teriparatide: ^‡^*p* < .05; ^‡‡^*p* < .01; ^‡‡‡^*p* < .001. Periosteal apposition at 0.2 to 0.6 mm from ridge; endosteal apposition at −0.2 to –0.6 mm from ridge. Abbreviations: BMC, bone mineral content; BMD, bone mineral density; Ct.Th, segmentation-based cortical thickness; SEM, standard error of the mean.

Resulting data for each treatment group are summarized in Tables 1 and 2. At Month 12, Ct.Th increased from baseline by (mean ± SD) 3 ± 6% (12 ± 21 μm in absolute terms; not significant) for placebo, 20 ± 13% (71 ± 46 μm; not significant vs placebo) for teriparatide, and 53 ± 18% (194 ± 66 μm; *p* < .001 compared with placebo and teriparatide) for romosozumab (or half these amounts if fully mineralized bone matrix was assumed; Figure 4A). With romosozumab treatment, cortical BMC (Figure 4B) and apparent cortical BMD (Figure 4C) were highly significantly increased from baseline (mean ± SD change from baseline: cortical BMC: 22 ± 5%; apparent cortical BMD: 47 ± 13 mg/cc; both *p* < .001 vs baseline), and in comparison to teriparatide (teriparatide: 11 ± 4% and 19 ± 6 mg/cc, respectively; both *p* < .001 vs romosozumab) and placebo (−1 ± 4% and −1 ± 7 mg/cc, respectively; both *p* < .001 vs romosozumab). These improvements were attained by both significant endosteal and periosteal bone matrix apposition, with greatest changes seen at the endosteal surface (Figure 4C). All other vertebral cortical bone standard variables demonstrated numerically greater changes with romosozumab treatment vs teriparatide and placebo, but did not all achieve statistical significance (Table 1).

**Table 1:** Vertebral cortical bone standard variables of Structural*Insight.*

Variable	Treatment group	Vertical cortex			Complete cortex		
		Baseline	Month 12	Change	Baseline	Month 12	Change
**BMD (mg/cc)** ^**a**^	Placebo	286 ± 51	290 ± 49	4 ± 11	294 ± 31	298 ± 29	4 ± 8
	Teriparatide	284 ± 31	312 ± 32	27 ± 14^††^	287 ± 32	307 ± 32	19 ± 8^††^
	Romosozumab	302 ± 41	365 ± 46^‡‡^	63 ± 20^‡‡‡^	307 ± 45	358 ± 50^‡‡^	51 ± 13^‡‡‡^
**Cortical thickness (Ct.Th [μm])**	Placebo	1329 ± 120	1299 ± 100	−30 ± 56	1418 ± 62	1390 ± 82	−27 ± 51
	Teriparatide	1259 ± 76	1330 ± 128	71 ± 71^††^	1350 ± 70	1415 ± 89	66 ± 75^†^
	Romosozumab	1267 ± 115	1365 ± 167	98 ± 92^††^	1336 ± 109^†^	1428 ± 148	92 ± 92^††^
**Density-weighted cortical thickness (wCt.Th [μm])**	Placebo	352 ± 59	350 ± 61	−2 ± 23	391 ± 42	389 ± 44	−2 ± 18
	Teriparatide	334 ± 47	389 ± 71	56 ± 32^††^	363 ± 46	407 ± 60	45 ± 22^††^
	Romosozumab	359 ± 58	477 ± 102^‡^	101 ± 51^‡‡‡^	389 ± 67	490 ± 105^‡^	101 ± 45^‡‡‡^
**Deconvoluted cortical thickness (dcCt.Th [μm])**	Placebo	366 ± 76	378 ± 73	12 ± 21	–	–	–
	Teriparatide	349 ± 49	420 ± 78	71 ± 46	–	–	–
	Romosozumab	364 ± 36	558 ± 77	194 ± 66^‡‡‡^	–	–	–

Values are mean ± SD.

a The initial segmentation was based on a template-driven, semi-automatic, active shape segmentation approach and represents an overall measure of BMD signals that reflect the underlying cortical, trabecular, and soft tissue structures as resolved by the CT imaging process. Significantly different from placebo: ^†^*p* < .05; ^††^*p* < .01; ^†††^*p* < .001; Significantly different from both placebo and teriparatide: ^‡^*p* < .05; ^‡‡^*p* < .01; ^‡‡‡^*p* < .001.

Abbreviations: μm, micrometers; BMD, bone mineral density; SD, standard deviation.

**Table 2:** Vertebral cancellous bone standard variables of Structural*Insight.*

Variable	Treatment Group	Spongiosa (2 mm peeled)			Subcortex (2 mm width)		
		Baseline	Month 12	Change	Baseline	Month 12	Change
**BMD (mg/cc)**	Placebo	95 ± 28	90 ± 32	−4 ± 5	143 ± 24	138 ± 26	−5 ± 4
	Teriparatide	99 ± 21	118 ± 25^†^	18 ± 10^†††^	144 ± 25	165 ± 30	21 ± 10^†††^
	Romosozumab	85 ± 23	105 ± 27	20 ± 7^†††^	131 ± 28	158 ± 34	27 ± 8^†††^
**TMD (mg/cc, above 250 mg/cc)** ^**a**^	Placebo	300 ± 8	300 ± 9	0 ± 4	326 ± 13	325 ± 13	−1 ± 5
	Teriparatide	314 ± 29	316 ± 26	2 ± 12	336 ± 27	337 ± 26	1 ± 10
	Romosozumab	329 ± 42^†^	336 ± 43^†^	7 ± 8	354 ± 45	366 ± 46^‡^	12 ± 10^‡‡^
**BV/TV (%, using 250 mg/cc)** ^**b**^	Placebo	7.5 ± 3.4	7.4 ± 3.8	−0.9 ± 0.9	16.9 ± 4.9	16.0 ± 5.4	−0.9 ± 1.5
	Teriparatide	11.1 ± 6.5	14.0 ± 7.6^†^	2.9 ± 3.9^†^	19.8 ± 7.9	24.3 ± 9.7^†^	4.5 ± 3.9^††^
	Romosozumab	12.4 ± 7.6	15.6 ± 8.3^†^	3.2 ± 2.8^†^	20.6 ± 7.7	26.6 ± 9.1^†^	5.9 ± 3.8^†††^

Values are mean ± SD.

a TMD was measured after first applying a threshold of 250 mg/cc to the data; the mean density of all voxels above the threshold was then computed within the trabecular segmentation.

b BV/TV was determined after first applying a threshold of 250 mg/cc to the data and represents the fraction of those voxels above the threshold divided by all voxels within the trabecular segmentation. Significantly different from placebo: ^†^*p* < .05; ^††^*p* < .01; ^†††^*p* < .001; Significantly different from both placebo and teriparatide: ^‡^*p* < .05; ^‡‡^*p* < .01; ^‡‡‡^*p* < .001.

Abbreviations: BMD, bone mineral density; BV/TV: total bone volume relative to total tissue volume; SD, standard deviation; TMD: tissue mineral density.

Improvements in cancellous BMD were similar between romosozumab and teriparatide (Table 2). All results were expressed in BMD for the following zones in order to compare the magnitude of bone apposition in specific vertebral compartments (Figure 4C): periosteal and endosteal compartments, both 0.4 mm thick with a shared boundary to a central cortical zone, also 0.4 mm thick; subcortical compartment, of ~2 mm thickness, bordering the endosteal compartment and peeled spongiosa compartment.

## Discussion

Numerous imaging techniques have previously been applied to evaluate changes in bone density and thickness in trials of romosozumab and teriparatide.^5^^,^^30^^,^^35–37^ This study contributes to the existing body of literature by demonstrating that using HR-QCT scans of the spine, it is possible to evaluate changes across the cortical shell of the vertebral bodies and determine changes in the endosteal and periosteal regions. The technique allowed evaluation of structural changes in the thin cortical shell of the vertebral body.

In postmenopausal women with low BMD, romosozumab treatment was associated with significantly greater gains in cortical thickness and improvement in all measured cortical parameters at Month 12 compared to treatment with teriparatide or placebo. In patients receiving romosozumab, these changes occurred through simultaneous endosteal and periosteal bone matrix apposition, with the greatest change seen at the endosteal surface.

Changes in evaluated cancellous bone parameters were similar in patients who received romosozumab and teriparatide. Trabecular, as well as subcortical BMD, were significantly increased by ~20% in both treatment groups at Month 12. BV/TV demonstrated a similar pattern, with increases of ~3% for the trabecular and ~5% for the subcortical region in both treatment arms. Only in romosozumab-treated patients was a change in TMD observed, which demonstrated a modest (~3%) increase in the subcortical region and was significantly different from both teriparatide and placebo. In contrast, no significant change was observed in any of the measured trabecular parameters, as expected.

In prior studies, romosozumab has similarly been shown to induce substantial changes in cortical thickness and BMD, measured via standard CT imaging, while also improving finite element analysis-estimated vertebral strength.^25^^,^^30^^,^^37^ Indeed, in the original phase 2 study of McClung et al., from which this study’s data are derived, romosozumab treatment was associated with an 11.3% increase in DXA-measured BMD at the lumbar spine, compared to a 7.1% increase and a 0.1% decrease with teriparatide and placebo, respectively.^5^ The improvements documented in the present study, of vertebral cortical BMD (at the endosteal, central, and periosteal compartments), subcortical BMD, and cortical thickness using this novel analytical technique, correspond well with those reported from the same patients, and on the same scans, using a 3D cortical bone mapping technique.^25^ In their study, using standard QCT scans, Poole et al. reported a 10.3% change from baseline in cortical thickness after 12 mo of romosozumab and a 4.3% increase with teriparatide.^25^ In the present study, romosozumab was associated with a 53% mean change from baseline in deconvoluted cortical thickness of the vertical cortex, compared to a 20% change with teriparatide. Using cortical maps, Poole and colleagues additionally demonstrated that with romosozumab treatment, the topographical locations of these changes occurred across the entire vertebra, including fracture-prone areas of the vertebral shell, walls, and endplates.^25^ However, Poole et al.’s method was not sufficiently sensitive to determine the presence or absence of periosteal apposition in response to treatments. Novelly, the present study expands upon Poole et al.’s observations by utilizing HR-QCT, which, in tandem with the application of the employed ICON methodology, was able to address the resolution-related limitations of standard QCT and better distinguish cortical from trabecular bone, detect microarchitecture changes, and identify endosteal, cortical and periosteal apposition. Thus, this study further adds to the existing literature by documenting the contributions from both the endosteal and periosteal cortical surfaces to the reported therapeutic changes in bone density and architecture.

It was previously reported that the large increase in cortical mass and thickness following romosozumab treatment was not accompanied by significant changes in external geometry of the spine (or hip), suggesting that the romosozumab-induced increase in cortical thickness was primarily the product of endosteal apposition.^35^ Those conclusions were limited, however, by the use of lower resolution QCT images processed using Medical Image Analysis Framework (MIAF) software (MIAF-Femur, version 5.8.0 M; MIAF-Spine, version 3.13; Institute of Medical Physics, University of Erlangen, Germany). Thus, the present study expands upon previous findings by demonstrating that both endosteal and periosteal bone matrix apposition contribute to gains in cortical thickness, BMC, and BMD at the spine with romosozumab.

The capacity for teriparatide treatment to stimulate improvements in cortical thickness in the vertebral body is more limited and may be negatively impacted by the pro-remodeling action of teriparatide, which could increase cortical porosity and endosteal remodeling.^25^^,^^38^ Indeed, some histomorphometric evidence suggests that teriparatide treatment increases trabecularization of the endocortical envelope.^39^ Our method does not allow for the determination of cortical porosity and, to our knowledge, the extent to which changes in cortical porosity may occur in the spine has never been documented in humans. If indeed these changes occur, they may be of limited magnitude due to the lower concentration of osteons in the vertebral cortical shell.^13^

Romosozumab treatment, on the other hand, has been shown to induce significant increases across the entire vertebra, including the cortical shell and endplates, which are also susceptible to fragility fractures.^25^ Indeed, while cortical bone constitutes only ~30% of the whole vertebrae,^40^^,^^41^ recent data suggests that reduction in vertebral cortical BMD has a greater impact on osteoporotic vertebral compression fractures than that of cancellous BMD.^42^ Furthermore, although trabecular bone handles much of the compressive forces, the cortex substantially contributes to overall vertebral strength, particularly in resisting bending and shear stresses. It has been proposed that vertebral cortical thickness may become a valuable measure for the prediction of osteoporotic fractures, but requires further exploration.^43^ Thus, the pan-vertebral increases in BMD and cortical thickness demonstrated in this study and others, suggests a means by which romosozumab treatment results in meaningful and rapid changes in reducing fracture risk, as previously reported.^8^

With growing evidence that osteoporosis negatively affects surgical outcomes (hardware failure) in patients undergoing spinal surgeries such as spinal fusion,^44^^,^^45^ these data may elicit interest to study the value of romosozumab in patients undergoing such procedures. Although, to date, no robust head-to-head comparison of the fracture risk-reducing effects of romosozumab vs teriparatide has been undertaken, together, these studies demonstrate that bone accrual with both romosozumab and teriparatide occurs in regions that are important for vertebral strength. Pertinently, however, the available data demonstrate that romosozumab treatment results in significant gains over other therapies, particularly in the cortical shell.

While not investigated in the current analysis, we have previously shown that the ICON algorithm drastically reduces the overestimation of cortical thickness seen with standard QCT scan data from more than 300% to 45% with low residual random accuracy errors.^20^ Indeed, while greater correlations were seen between HR-QCT-measured and HR-pQCT-measured cortical thickness, good performance vs the gold standard was nevertheless achieved by applying the ICON algorithm to standard QCT. As such, the method employed may be of additional value for limiting radiation exposure.

Technological advances in hardware and software are constantly improving image quality in computed tomography. The introduction of techniques such as dual-energy and photon counting detectors in conjunction with new software paradigms like AI-based reconstruction algorithms are driving rapid changes, which are bringing HR-QCT-level data quality to clinical practice with comparable radiation exposure as traditional standard (Q)CT protocols. This opens new opportunities for the application of deconvolution-based methods like ICON and its more robust estimation of the dcCt.Th thickness parameter, which becomes more accurate with increased image resolution.

### Limitations

One limitation of this study is the limited sample size in each treatment group. Nevertheless, the study was of sufficient statistical power to detect significant between-group differences in compartmental changes in the vertebral cortical shell. Furthermore, the findings of this study corroborate those of a prior study utilizing a different technique to assess the same images.^25^ Altogether, these findings provide a robust indication of strength reinforcement of the vertebrae with new bone in response to bone-forming therapies. The precise relative contribution of cortical bone to vertebral body strength and its significance in the reduction of fracture risk requires further investigation.

By applying the ICON method to HR-QCT data, we report a robust estimation of changes in cortical thickness, expanding upon previous evidence of the accuracy of such measurements by demonstrating sufficient sensitivity to detect significant differences in response to osteoporosis treatments, through estimating the thickness of the vertical portion of the cortex and thus neglecting, for instance, the endplates or pedicles, to minimize the potential negative influence of poor segmentation given the CT data’s resolution.^20^ We acknowledge that differentiation between endosteal and periosteal cortex is prone to disturbances due to the segmentation process. In these analyses, changes in cortical thickness were modeled assuming an average of 50% mineralization of newly added matrix, based on the presumption of ongoing anabolic bone gain during the 12-mo period. Thus, if mineralization was in fact ≥50%, changes in cortical thickness would be overestimated. Nevertheless, even at 100% mineralization, which would result in changes of half the magnitude described herein, these results demonstrate treatment effects.

Another limitation of the study is related to the unknown exact effect of, or differences in, precise mineralization changes in the cortex with both therapies. However, there is no reason, a priori, to expect that changes in mineralization would significantly differ between teriparatide and romosozumab administration. Future collection of photon-counting CT data with increased resolution and signal-to-noise ratio, in conjunction with the ICON method, will provide further details regarding the precise location of mineralization changes during treatment. In this study, an assumed, physiologically relevant, fixed mineralization of 1100 mg/cc was used.^33^ While alternative mineralization densities were not applied to our analyses, modulation of the assumed mineralization would convey a linear scaling effect to wCt.Th (e.g. a 20% higher assumed mineralization density would elicit a 20% decrease in estimated wCt.Th) within a useful physiological range (e.g. 900 to 1200 mgHA/cc), while a nonlinear effect may occur (for better or worse) in the ICON analysis; further research would be required to explore this in more detail.

At the resolution employed in this study, it was not possible to assess cortical porosity. However, given the small thickness of the cortex and the very limited number of osteons,^13^ we did not expect substantial bone-forming effects to be mediated by a reduction in porosity in the cortex present at baseline. Finally, while the present study provides methodologically robust evidence of greater improvements in cortical parameters after 12 mo of romosozumab treatment vs teriparatide, further research is required to elucidate comparative efficacy over the longer term.

## Conclusions

These data provide mechanistic evidence supporting the effect of romosozumab to increase bone formation as a beneficial therapeutic option that significantly improves cortical bone parameters within 12 mo. While the observed changes in cancellous bone parameters were similar with romosozumab and teriparatide treatment, romosozumab significantly increased changes in cortical BMC and apparent cortical BMD from baseline, and compared to teriparatide, which was attained by both endosteal and periosteal bone matrix apposition. The location and magnitude of these changes likely form the basis of, and explain, the rapid improvement in bone mass, structure, and strength that contribute to the rapid reduction in the risk of vertebral fractures reported with romosozumab treatment across all vertebral fracture grades.^6^^,^^7^

## Supplementary Material

HR_QCT_Manuscript_Supplementary_Material_18April24_ziaf119

## Data Availability

Qualified researchers may request data from Amgen clinical studies. Complete details are available at the following: http://www.amgen.com/datasharing.
