# Supplementary material for: Romosozumab significantly improves vertebral cortical bone mass and structure compared with teriparatide, whereas both treatments increase vertebral trabecular bone mass similarly: high-resolution quantitative computed tomography analyses of randomized controlled trial results in postmenopausal women with low bone mineral density
Source: JBMR Plus. 2025 Jul 15;9(10):ziaf119. doi: 10.1093/jbmrpl/ziaf119 (PMC12449578; doi:10.1093/jbmrpl/ziaf119)
Supplement: HR_QCT_Manuscript_Supplementary_Material_18April24_ziaf119 [file hr_qct_manuscript_supplementary_material_18april24_ziaf119.docx]

**­Supplementary Material**

Supplementary Material 1. Mathematical principles of the deconvolution model used in the Iterative Convolution OptimizatioN (ICON) method.

The following description is adapted from our earlier methodological paper, which contains a comprehensive description of the ICON method.^(1)^

Rationale and concept

Image convolution is a standard approach for modeling spatial resolution in the context of the imaging process. The dataset is folded with a kernel functional by means of spatial integration. A Gaussian functional is commonly used to model the blurring caused by limited spatial resolution, also incorporating noise. For CT data, filter kernel differs for in-plane variations versus those observed in the z-direction. In single-slice CT, one could use a step function in
z-direction, but considering commonly available fan-beam and helical CT data, this simplification is not justified anymore.

The ICON method developed for improving blurring effects in CT imaging of bone is based on a deconvolution approach, in which the convolution effects during data acquisition are estimated and corrected for. In this appendix we describe the different parts of our convolution kernel, used to generate a combined filter, which in turn was used in a standard nonlinear solver (JMP 9.0, SAS Institute, Cary, NC, USA) to estimate the model parameters of the modeling process that describes the imaging process. This solving process can be considered as a deconvolution, as we backwards calculate the imaging process. Direct deconvolution of 2D/3D data suffers from substantial sensitivity to noise leading to instability. Our method, which is iterative in nature, is very stable, as it works on the averaged data of our radial density distributions calculated for an initial 3D segmentation. Constraining the combined filter to the assumed model of the spongiosa-cortex-soft tissue background complex further stabilizes the procedure.

Mathematical modeling

**Point Spread Function (PSF) and image convolution**

We assume a Gaussian imaging filter kernel. As we work just on the vertical cortex, the complex 3D problem is reduced to 2D:

$$PSF \left( x,y, \sigma\right)=\frac{{e^{-}}^{\frac{x^{2}+y^{2}}{{2\sigma}^{2}}}}{{2\pi\sigma}^{2}}$$

with in-plane coordinates *x* and *y* and the variance *σ^2^*, where the full-width-half maximum FWHM(*σ*)is given as:

$$2\sqrt{{2\sigma}^{2}\cdot ln2} \approx2.355\cdot\sigma$$

Following the approach reported by Prevrhal,^(2)^ the radially symmetric σ is assumed to define the complete imaging process (performance depending on scanner type, scan protocol, and CT reconstruction kernel). The process of image convolution to transfer the object geometry into the

resulting CT data is derived by integration:

$$CT Data (x, y) =\iint BMD\_Model (x, y)\cdot PSF (x-x', y-y')dx'dy'$$

**Edge Spread Function (ESF)**

Similarly, the Edge Spread Function (ESF) used to model one side of an infinite wide step function:

$$ESF \left( x,y,\sigma,bmd,x_{0} \right)=\int_{-\infty}^{x_{0}} \int_{-\infty}^{+\infty} bmd\cdot PSF\left( x-x^{'},y-y^{'} \right)dx'dy'$$

with constant density plateau height *bmd* and step position *x_0_* being deduced by convolution. In our model it was used to model the influence of the mean BMD of the spongiosa inside the vertebrae, which, after convolution, also spreads into the cortex region and generates a shift of the radial density distribution peaks inwards. Another ESF has been used to model the soft tissue signal from outside the vertebra.

**Line Spread Function (LSF)**

To model the thin cortex of width *d*, a similar convolution was made to deduce the Line Spread Function (LSF) as follows:

$$LSF \left( x,y,\sigma,{bmd}_{full},x_{0},w \right)=\int_{x_{0}}^{x_{0}+w} \int_{-\infty}^{+\infty} {bmd}_{full}\cdot PSF\left( x-x^{'},y-y^{'} \right)dx'dy'$$

with constant *bmd_full_* = e.g. 1,100 mgHA/cm^3^, center position *x_0_* and width *w*.

**Combined Model**

To apply a single function to the collected radial density distribution of the ICON method, we just had to combine one LSF reflecting the cortex with two ESF for the bones interior trabecular network and the soft tissue outside the bone with adopted parameters to form a continuous model of the assumed bone profile.

$$Combined Model \left( \sigma,{bmd}_{spongiosa},{bmd}_{full},{bmd}_{soft\_tissue,}x_{0},w \right)={ESF}_{spongiosa}\left( \sigma,{bmd}_{spongiosa},x_{0} \right)+{LSF}_{cortex}\left( \sigma,{bmd}_{full},x_{0},w \right)+{ESF}_{Soft\_Tissue}(\sigma,{bmd}_{soft\_tissue},x_{0}+w)$$

Using this combined model in a numerical solver, e.g. nonlinear fit of JMP 9.0 (SAS Institute, Cary, NC, USA), using fixed σ and *bmd_full_*, the remaining four parameters *bmd_spongiosa_*, *bmd_soft_tissue_*, endosteal cortex edge *x_0_* and width *w*, referred to as deconvolved cortical thickness in this paper, can be solved at once.

Due to the pronounced profile shape after averaging the data in the layers, this solving procedure is very stable. Using prominent contrast edges, e.g. from (untreated) cortices, scanner-/reconstruction-kernel-specific σ parameters, which reflect the complete imaging and analysis procedure by means of sharpness/blurriness, can be estimated.

We are open to share the code of the ICON method with interested researchers.

**References**

1. Damm T, Peña JA, Campbell GM, Bastgen J, Barkmann R, Glüer C-C. Improved accuracy in the assessment of vertebral cortical thickness by quantitative computed tomography using the Iterative Convolution OptimizatioN (ICON) method. Bone. Mar 2019 2019;120:194-203.
2. Prevrhal S, Engelke K, Kalender WA. Accuracy limits for the determination of cortical width and density: the influence of object size and CT imaging parameters. Phys Med Biol. Mar 1999;44(3):751-64.
